# In Patients with Dysimmune Motor and Sensorimotor Mononeuropathies, the Degree of Nerve Swelling Correlates with Clinical and Electrodiagnostic Findings

**DOI:** 10.3390/neurosci7010005

**Published:** 2026-01-03

**Authors:** Simon Podnar

**Affiliations:** 1 Institute of Clinical Neurophysiology, Division of Neurology, University Medical Center Ljubljana, 1000 Ljubljana, Slovenia; simon.podnar@kclj.si; 2 The Faculty of Medicine, University of Ljubljana, 1000 Ljubljana, Slovenia

**Keywords:** electrodiagnosis, inflammatory mononeuropathy, nerve cross-sectional area, peripheral nerves, ultrasonography

## Abstract

In non-vasculitic immune-mediated neuropathies, imaging studies demonstrate an enlargement not only of clinically involved but also of clinically intact nerves. The present study aimed to present a pattern of nerve swelling and its relation to nerve function. In a group of patients with dysimmune motor and sensorimotor mononeuropathies, nerve cross-sectional areas (CSAs) were measured using ultrasonography (US) and compound muscle action potential (CMAP) amplitudes using electrodiagnostic (EDx) studies. Nerve CSAs were compared in (1) clinically involved, (2) swollen and clinically uninvolved, and (3) non-swollen (clinically uninvolved) nerves. Patients’ non-swollen nerves were also compared to those of controls. In swollen nerves, the correlation between nerve CSA and CMAP amplitude was calculated. Twenty-two patients (12 men) and 50 controls (28 men) were included in the study. Clinically involved nerves were thicker than swollen segments of clinically intact nerves (*p* < 0.001). The patients’ non-swollen (clinically uninvolved) nerves were thicker than the controls’. In swollen nerves, CSA was strongly negatively correlated with CMAP amplitude (r = −0.54, *p* < 0.001). In patients with immune-mediated mononeuropathies, nerve swelling correlates with clinical and EDx findings. Patients’ clinically uninvolved nerves were also swollen, but to a lesser degree.

## 1. Introduction

Currently, there is no accepted description of the mononeuropathic variant of multifocal motor neuropathy (MMN) and multifocal chronic inflammatory demyelinating polyneuropathy (CIDP). However, the latter can be included under the focal CIDP variant [1]. To diagnose a dysimmune mononeuropathy, first, unexplained clinical involvement of a single peripheral nerve should be demonstrated and confirmed by electrodiagnostic (EDx) studies. Second, using imaging studies, nerve swelling at a non-entrapment site or unusually long nerve swelling at the entrapment site should be demonstrated. Similar to MMN and multifocal CIDP, dysimmune mononeuropathies predominantly affect the upper limbs. Cerebrospinal fluid (CSF) analysis, antiganglioside antibodies, and response to immune therapy can support the diagnosis in a proportion of dysimmune mononeuropathy patients [2]. Laboratory investigations can also help to exclude other diagnoses (e.g., vasculitic neuropathies).

A diagnosis of probable dysimmune motor mononeuropathy (MM) and sensorimotor mononeuropathy (SMM) is also supported by ultrasonographic (US) findings of nerve swelling of several otherwise clinically intact nerves previously described in MMN [3]. In this author’s previous study, a median of six swollen, clinically intact nerves was found in MM and SMM [2]. Similar to MMN and multifocal CIDP, their mononeuropathic variants are therefore also presumably caused by an immune-mediated process targeting not only clinically involved but also clinically intact nerves.

The clinical, EDx, and US features of MM and SMM patients, and their response to treatment, have already been described [2]. Here, a detailed quantitative analysis of nerve cross-sectional areas (CSAs) in MM and SMM is reported. This analysis was used to test the hypothesis that dysimmune (multiple) mononeuropathies are due to a generalized immunological process, causing the most severe nerve swelling in clinically involved nerves, followed by swollen clinically intact nerves, and finally non-swollen clinically intact nerves.

To test this hypothesis, a post hoc analysis of data obtained in a previously reported prospective study of MM and SMM patients was performed, comparing nerve CSAs of (1) clinically involved and (2) swollen clinically intact nerves. Patients’ swollen nerve CSAs were correlated to their EDx parameters. Additionally, the non-swollen nerve CSAs of patients were compared to those of control subjects.

## 2. Materials and Methods

### 2.1. Patients and Controls

As in a previous study [2], electronic medical charts were reviewed to identify MM or SMM patients. Inclusion criteria were (1) clinical and EDx signs of mononeuropathy not explained by other causes, (2) no clinical or EDx involvement of additional peripheral nerves for at least 12 months after the onset of mononeuropathy symptoms, and (3) US thickening of the peripheral nerves at non-entrapment sites, or ≥ 10 cm long nerve US thickening at the entrapment site [2]. Exclusion criteria were (1) entrapment or compression neuropathies, (2) nerve trauma, (3) vasculitic neuropathy (excluded mainly on clinical and laboratory grounds [4]—nerve biopsy was not performed), (4) neuralgic amyotrophy (excluded on clinical grounds including the pattern of nerve involvement [5]), (5) diabetic radiculo-plexus-neuropathy, (6) hereditary neuropathy with liability to pressure palsies (excluded on clinical and EDx grounds), (7) peripheral nerve tumors (excluded on clinical and imaging findings—including MR), and (8) motor neuron disorders including monomelic amyotrophy (excluded on clinical and EDx grounds) [2]. The National Ethics Committee of Slovenia approved the study (No. 0120-184/2020/3), and before their inclusion, written informed consent was obtained from all patients and controls.

### 2.2. History and Clinical Neurologic Examination

The author obtained a history and, on the same day, performed clinical neurologic examinations, EDx, and US studies in all patients as previously described [2]. He was not blinded to patients’ clinical conditions and findings from previous steps of the evaluation.

Patient sex, age at the onset of symptoms, duration of symptoms, characteristics of symptoms, treatment, and concomitant disorders were obtained from patients’ histories, supplemented by electronic medical records. During neurological examination, muscle atrophy and the strength of all principal limb muscle groups were graded [6]. On all limbs, touch and pinprick sensation of the principal skin areas was graded using cotton wool and a pin. The strength of the most distal muscles and sensation on the skin innervated by the involved nerve were included in analyses.

### 2.3. Electrodiagnostic Studies

Bilateral motor and sensory nerve conduction studies (NCSs) of the main upper limb nerves (median, ulnar, and radial), and one lower limb (usually the left: fibular, tibial, superficial fibular, and sural) were performed using standard commercially available EMG equipment (Nicolet Synergy, Natus Medical Incorporated, San Carlos, USA). During motor NCSs, in addition to distal stimulation (terminal distance: 8 cm), and stimulation at the elbow/knee, nerves were also stimulated at more proximal sites (ulnar in the upper arm and at the brachial plexus, radial nerve at the upper arm). During antidromic sensory NCSs, the distance between stimulation and recording sites was 14 cm, except for the superficial fibular nerve (8 cm). Terminal motor latencies, compound muscle action potential (CMAP) and sensory nerve action potential (SNAP) baseline-to-peak amplitudes, motor and sensory nerve conduction velocities, and F-wave latencies were measured. Measured parameters were compared to our laboratory’s NCS reference values. Needle electromyography (EMG) of at least one distal muscle innervated by a clinically and EDx-involved peripheral nerve was also performed.

Clinically involved nerves were defined by (1) muscle atrophy or weakness (MRC score 4 or less) and/or (2) reduced sensation in the appropriate distribution, supported by EDx findings for the involved nerve: (1) CMAP amplitude below the lower reference limit, (2) motor nerve conduction block of 30% or more, or (3) SNAP amplitude below the lower reference limit.

### 2.4. Ultrasonography (US)

Bilateral US studies of the main upper limb nerves (median, ulnar, and radial) and nerves of one lower limb (usually the left: fibular, tibial, and sural) were performed using standard commercially available US equipment (Sequoia, Siemens Healthineers, Erlangen, Germany) and an Acuson 14-5-MHz linear array transducer. Nerve CSAs were measured with a US probe perpendicular to the assessed nerve, using a trace method excluding the hyperechoic nerve rim [7]. CSA was measured at standard sites or segments: (1) median and ulnar: wrist, forearm, elbow, upper arm; (2) radial: elbow, upper arm; (3) fibular: fibular head, popliteal fossa; (4) tibial: ankle, popliteal fossa; (5) sural nerve: calf. The nerves were scanned along the whole segments (e.g., the median nerve in the forearm), and CSA was also measured at the point of maximal thickness. Measured nerve CSAs were compared to our set of reference values [8].

Data obtained from a group of previously reported control subjects was used for comparison [8]. In this group, US studies were performed using another US system (ProSound Alpha 7, Hitachi Aloka Medical, Ltd., Tokyo, Japan) and a 4–13 MHz linear array transducer. In control subjects, US studies were performed unilaterally, and radial nerve studies were not performed [8].

### 2.5. Statistics

**Figure 1:**
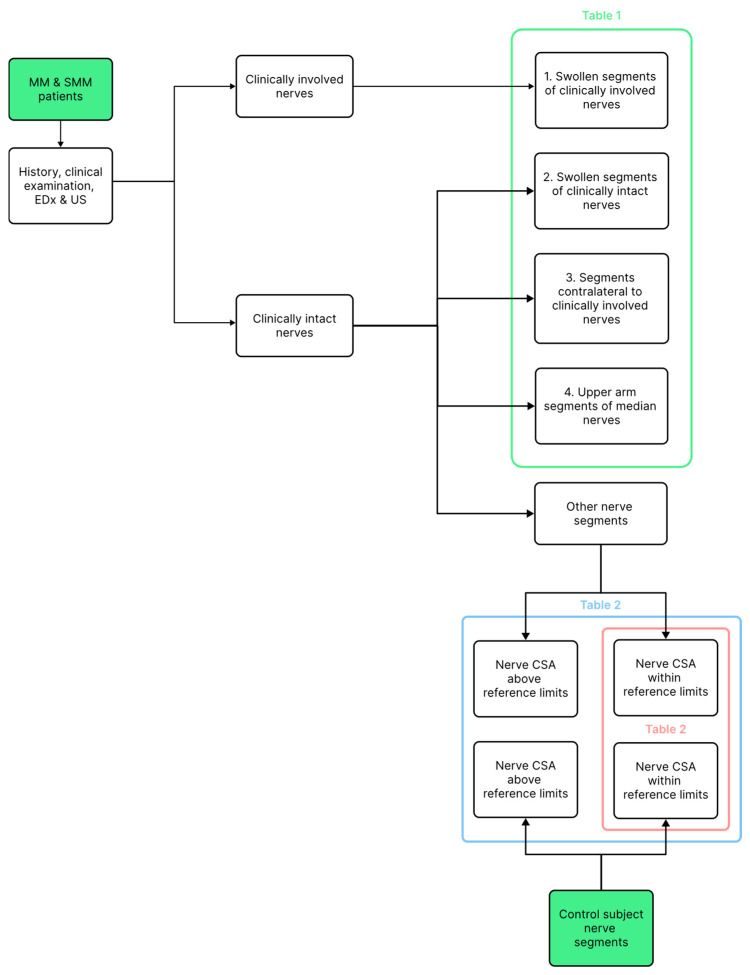
A schematic diagram illustrating the study framework.

Data were entered into a standard spreadsheet (Excel, Microsoft, Redmond, WA, USA). Patients’ CSAs of all nerves (except the radial) measured on standard sites were compared to control nerve CSAs [8]. This comparison was repeated after excluding all nerve segments with CSA above the upper reference limit (Figure 1). For swollen segments of clinically involved nerves, swollen clinically uninvolved nerves, nerves contralateral to clinically involved nerves, and median nerves in the upper arm, the ratio of the patients’ CSA to the control mean CSA for that nerve segment reported in a recent meta-analysis [9] was calculated. Likewise, for nerves with swollen segments, the ratio of the patients’ CMAP amplitudes on proximal stimulation to the control mean CMAP amplitudes obtained from our reference data was calculated. As all CSA and CMAP amplitude values and their ratios were distributed non-normally (as established by the Shapiro–Wilk test), non-parametric descriptive statistics (i.e., median (range or 25th–75th percentile)), and for comparisons, non-parametric tests (i.e., the Mann–Whitney and the Kruskal–Wallis test) were used. In swollen nerves, Pearson’s correlation coefficient (r) was calculated between nerve CSA and CMAP amplitude ratios. All statistical analyses were performed using the Statistics Kingdom [10], an online statistical package. All tests were performed at a significance level of α = 0.05 (two-sided).

## 3. Results

The studied population included 22 patients (12 men, 56%): 12 patients (7 men) with SMM and 10 patients (5 men) with MM, as reported previously [2]. The patient’s age at the onset of neurological symptoms varied from 34 to 72 years (median, 48 years). During follow-up examination, symptoms had been present for 2 months to 20 years (median, 2.3 years). The most commonly clinically involved nerves were the ulnar (14 patients), followed by radial (6 patients) and median (2 patients) [2]. Five MM patients were treated with immunoglobulins, four long-term, and two of them by subcutaneous infusions. In one MM patient, rituximab was added to immunoglobulins. Patients with SMM were treated with intravenous corticosteroids (three patients), oral corticosteroids (two patients), and, in one patient, intravenous immunoglobulins were also added [2]. The control group included 50 subjects (28 men, 54%), aged from 18 to 66 years (median, 31 years) [8]. Patients’ age was significantly higher than the controls’ age (*p* < 0.01).

Significant differences in CSAs between four evaluated nerve segments (Figure 1) were found (*p* < 0.001, effect size η^2^ was 0.32 (large)). Clinically involved nerve segments were thicker than all three other evaluated segments (Table 1). No difference between CSA ratios of clinically involved swollen nerve segments of treated and untreated patients was found (median CSA ratio: 2.00 and 1.39, respectively; *p* = 0.26). Ratios of swollen segments of clinically normal nerves were similar to those of nerves contralateral to the involved nerves (*p* = 0.17) but were thicker compared to median nerves in the upper arm (<0.01).

**Figure 2:**
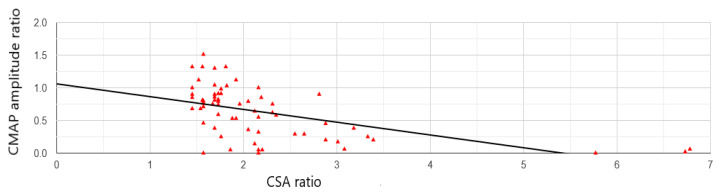
Pearson’s correlation coefficient (r) between swollen nerve cross-sectional area (CSA) ratio and compound muscle action potential (CMAP) amplitude on proximal stimulation ratio (r = −0.54, *p* < 0.001). To be pooled, median, ulnar, and radial nerves were normalized by dividing their CSAs and CMAPs by their corresponding mean values obtained in controls [9].

In clinically involved nerves, CMAP amplitudes on proximal stimulation varied from 0 to 12 mV (median 2.3 mV), and maximal CSAs from 8 to 40 mm^2^ (median 14 mm^2^). In swollen clinically uninvolved nerves, CMAP amplitudes on proximal stimulation varied from 0.3 to 14.9 mV (median 8.0 mV), and maximal CSAs from 8 to 17 mm^2^ (median, 12 mm^2^). After normalization, maximal CSAs were negatively correlated with CMAP amplitudes (r = −0.54, *p* < 0.001, Figure 2).

Even after excluding all swollen nerves, the patient’s upper limb nerves were thicker than those of controls (Table 2). The only exceptions were the upper arm segment of the ulnar nerve, which was thicker in controls, and the elbow segment of the ulnar nerve, with no difference. The thickening of the patient’s lower limb nerves was less consistent, both before and after excluding the swollen nerve segments (Table 2).

## 4. Discussion

The present study demonstrated a generalized thickening of peripheral nerves in patients with immune-mediated mononeuropathies. This finding was unchanged even after excluding all swollen nerves. This supports the hypothesis that, despite the clinical involvement of only a single nerve, in patients with dysimmune mononeuropathies, all peripheral nerves are involved. Similar thickening of clinically uninvolved peripheral nerves was previously demonstrated in neuralgic amyotrophy [11], another dysimmune (multiple) mononeuropathy. Such generalized thickening of nerves was found predominantly in the upper limbs, which follows the predilection of focal dysimmune neuropathies (e.g., MMN and multifocal CIDP) for upper limb nerves. The largest difference was observed in the upper arm segment of the median and the forearm segment of the ulnar nerve.

The study also supported the hypothesis that in SMM and MM patients, clinically involved nerves are thicker compared to swollen clinically uninvolved nerves, nerves contralateral to involved nerves, and the median nerves in the upper arm. This finding supports the view that in segments with more severe nerve swelling, nerve function is impaired, leading to clinical symptoms, abnormalities on neurological examination, and abnormal EDx findings. In nerves with less severe swelling, nerve function remains preserved. It is well-documented that with time, in some patients with dysimmune mononeuropathies, additional nerves become clinically involved [12,13], thus fulfilling diagnostic criteria of probable MMN or multifocal CIDP [1,14]. However, no prospective US data have been published demonstrating an increase in nerve swelling during the transition of nerves from clinically intact to clinically involved.

The relation between nerve morphology and function was, in the present study, substantiated by the demonstration of a negative correlation between the nerve CSA and CMAP amplitude; larger nerve swelling was associated with lower CMAP amplitudes. In CIDP, the correlation between CSA and CMAP amplitude was sought only in a single previous study, but no correlation was found [15]. That study calculated correlation with distal CMAP amplitudes, while in the present study, CMAP amplitudes obtained on proximal stimulation were used. Proximal stimulation includes not only CMAP reduction due to axonal loss, but also conduction block and temporal dispersion in the intervening nerve segment. This might raise the sensitivity of this parameter. In MMN, colocalization between nerve CSA and conduction block was found in 9 of 36 (25%) sites [16]. In another CIDP study, segmental nerve enlargements were often seen at sites of conduction block or temporal dispersion [17]. Previous CIDP studies have shown a weak to moderate correlation between CSA and motor conduction velocity [15,17,18]. Conduction velocity is important for the diagnosis of nerve demyelination, but does not directly translate to nerve clinical involvement (e.g., muscle atrophy and weakness). However, it is often associated with it. To explore the relation between CSA and motor conduction velocity, future studies should try to measure conduction velocity also in the proximal nerve segments.

Nerves contralateral to clinically involved nerves are typically also swollen, as demonstrated by the similar CSA of these nerves compared to swollen clinically normal nerves. This suggests that in immune-mediated mononeuropathies, immunologic attack might be partially specific for individual peripheral nerves, e.g., ulnar, radial, or median. This feature is also reflected in the different frequencies of involvement of these nerves [2].

In CIDP and its variants [19], as well as in MMN [3,20], for unclear reasons, the median nerves in the upper arm are preferentially involved. This nerve segment was therefore also added to the analysis. However, swollen clinically normal nerves were thicker, probably due to the swelling of the upper arm median nerve segment in some but not in all patients’ arms.

Even in control subjects, CSAs of the three main upper limb nerves on different sites/segments vary considerably. Therefore, to pool them, their CSA had to be normalized, i.e., the ratio of the measured CSA was divided by the control mean CSA. This had to be performed for swollen nerve segments (clinically involved and uninvolved), nerves contralateral to involved nerves, and median nerves in the upper arm. By contrast, normalization was not needed for the comparison of patients’ and controls’ nerves, where their corresponding nerve segments were compared.

Comparison of patients’ and controls’ nerves after the exclusion of all swollen nerve segments was aimed at answering the question of whether all peripheral nerves are involved in dysimmune mononeuropathies. This analysis confirmed slight generalized peripheral nerve swelling (i.e., involvement) in SMM and MM patients.

Five MM and SMM patients were treated, mainly with immunoglobulins and corticosteroids, respectively, which increased their heterogeneity. These treatments were expected to reduce EDx and US abnormalities (i.e., increase CMAP amplitudes and reduce nerve CSAs), driving the differences closer to the null hypothesis. However, no difference between CSA ratios of clinically involved swollen nerve segments of treated and untreated patients was found.

This study has several limitations. Firstly, the number of included patients is relatively small. Nevertheless, several of the compared variables demonstrated statistical significance. Secondly, the control subjects were younger compared to the patients. However, a recent meta-analysis did not reveal a significant correlation between age and nerve CSA in control subjects [9]. Thirdly, different examiners performed CSA measurements in patients and controls using different commercially available US systems, which may have introduced variability. However, in a recent meta-analysis, substantial heterogeneity was found only for the radial nerve [9], which was not included in the patients vs. controls comparison. Additionally, during the US examination, the author was not blinded to the clinical features and EDx findings of patients. However, at that time, this study’s hypothesis had not been formed. Last but not least, a 10 cm length was applied as an inclusion criterion, although this is not a validated cut-off but rather based on the finding that in focal neuropathies due to external compression or entrapment, the affected segment is usually less than 10 cm long [21].

## 5. Conclusions

The present study supports the generalized involvement of peripheral nerves in immune-mediated mononeuropathies. Clinically involved nerves are more severely swollen compared to clinically intact swollen nerves. The degree of swelling correlates with CMAP amplitude. Further studies are needed to verify an increase in nerve swelling on transition from clinically uninvolved to clinically involved, i.e., from MM to MMN, and SMM to multifocal CIDP.

## Figures and Tables

**Table 1:** Comparison of nerve cross-sectional area (CSA) ratios in 22 patients with non-vasculitic immune-mediated mononeuropathies.

	Number	Nerve CSA Ratio Median (Range)	*p*-Value: * < 0.01, ** < 0.001
1. Clinically involved nerve segments	21	2.35 (1.54–6.78)	1 vs. 2 *, 1 vs. 3 **
2. Swollen clinically uninvolved nerve segments	18	1.76 (1.56–2.71)	2 vs. 3 = 0.17,
3. Nerves contralateral to the involved nerves	20	1.62 (0.91–4.07)	3 vs. 4 = 0.21
4. Median nerves in the upper arm	42	1.45 (0.96–2.05)	1 vs. 4 **, 2 vs. 4 *

Nerve segments named in the first column are compared in the last column.

**Table 2:** Comparison of nerve cross-sectional areas (CSAs) of all and non-swollen nerve segments of 22 patients with non-vasculitic immune-mediated mononeuropathies, and 50 control subjects.

	All Nerve Segments					Non-Swollen Nerve Segments				
	Patients		Controls			Patients		Controls		
	*N*	Median (25–75 Perc.)	*N*	Median (25–75 Perc.)	*p*-Value	*N*	Median (25–75 Perc.)	*N*	Median (25–75 Perc.)	*p*-Value
Median wrist	44	13 (11–15.25)	50	10 (8–11.75)	**<0.001**	12	11 (10–11)	37	9 (8–10)	**<0.001**
Median forearm	44	8 (7–9)	50	7 (6–8)	**<0.001**	38	7.50 (7–8.75)	47	6 (6–8)	**0.002**
Median elbow	44	12 (10–14)	50	11.50 (10–13)	**<0.001**	42	12 (10–14)	48	11 (10–13)	**<0.001**
Median upper arm	44	11.50 (10–13)	50	7 (6–8)	**<0.001**	22	11 (9–11)	49	7 (6–8)	**<0.001**
Ulnar wrist	43	7 (6–8)	50	6 (5–7)	**0.008**	43	7 (6–8)	49	6 (5–7)	**0.004**
Ulnar forearm	42	9 (8–9.75)	50	6 (5–7)	**<0.001**	31	8 (7–9)	49	6 (5–7)	**<0.001**
Ulnar elbow	43	11 (8.50–12.50)	50	8 (7–9.75)	**<0.001**	21	8 (8–9)	44	8 (7–9)	0.38
Ulnar upper arm	43	8 (7–10)	50	10 (9–11)	**<0.001**	30	7.50 (7–8)	23	9 (8–9)	**<0.001**
Fibular head	20	11 (10–12.50)	48	10 (8–12)	**0.038**	15	11 (9.50–11)	45	10 (8–11)	**0.02**
Fibular knee	20	8 (6.75–9)	48	7 (6–9.25)	0.41	20	8 (6.75–9)	44	7 (6–8)	0.10
Tibial ankle	16	13 (12–13.25)	48	11 (9–13.25)	0.053	16	13 (12–13.25)	46	11 (9–12.75)	**0.015**

25–75 perc—25th–75th percentile; median (25–75 perc.) is given in mm^2^. Significant *p*-Values are shown in bold.

## Data Availability

The raw data supporting the conclusions of this article will be made available by the author on request.

## Abbreviations

The following abbreviations are used in this manuscript:

CIDP	Chronic Inflammatory Demyelinating Polyneuropathy
CMAP	Compound muscle action potential
CSA	Cross-sectional area
CSF	Cerebrospinal fluid
EDx	Electrodiagnostic
EMG	Electromyography
MM	Motor mononeuropathy
MMN	Multifocal motor neuropathy
MRC	Medical Research Council
NCSs	Nerve conduction studies
SMM	Sensorimotor mononeuropathy
SNAP	Sensory nerve action potential
US	Ultrasonography
